# Predictability of the effects of facet joint infiltration in the degenerate lumbar spine when assessing MRI scans

**DOI:** 10.1186/s13018-017-0685-x

**Published:** 2017-11-21

**Authors:** Ulf Krister Hofmann, Ramona Luise Keller, Christian Walter, Falk Mittag

**Affiliations:** 1 Department of Orthopaedic Surgery, University Hospital of Tübingen, Hoppe-Seyler-Strasse 3, 72076 Tübingen, Germany; 2 Faculty of Medicine, Julius-Maximilians University of Würzburg, Josef-Schneider-Str.2, 97080 Würzburg, Germany

**Keywords:** Lumbar spinal stenosis, Lumbar degenerative disease, MRI, Facet joint degeneration, Facet joint injection

## Abstract

**Background:**

Imaging results are frequently considered as hallmarks of disease by spine surgeons to plan their future treatment strategy. Numerous classification systems have been proposed to quantify or grade lumbar magnetic resonance imaging (MRI) scans and thus objectify imaging findings. The clinical impact of the measured parameters remains, however, unclear. To evaluate the pathological significance of imaging findings in patients with multisegmental degenerative findings, clinicians can perform image-guided local infiltrations to target defined areas such as the facet joints.

The aim of the present retrospective study was to evaluate the correlation of MRI facet joint degeneration and spinal stenosis measurements with improvement obtained by image-guided intraarticular facet joint infiltration.

**Methods:**

Fifty MRI scans of patients with chronic lumbar back pain were graded radiologically using a wide range of classification and measurement systems. The reported effect of facet joint injections at the site was recorded, and a comparative analysis performed.

**Results:**

When we allocated patients according to their reported pain relief, 27 showed no improvement (0–30%), 16 reported good improvement (31–75%) and 7 reported excellent improvement (> 75%). MRI features assessed in this study did, however, not show any relevant correlation with reported pain after facet joint infiltration: Values for Kendall’s tau ranged from *τ* = − 0.190 for neuroforaminal stenosis grading as suggested by Lee, to *τ* = 0.133 for posterior disc height as proposed by Hasegawa.

**Conclusion:**

Despite the trend in evidence-based medicine to provide medical algorithms, our findings underline the continuing need for individualised spine care that, along with imaging techniques or targeted infiltrations, includes diagnostic dimensions such as good patient history and clinical examination to formulate a diagnosis.

**Trial registration:**

ClinicalTrials.gov, NCT03308149, retrospectively registered October 2017

**Electronic supplementary material:**

The online version of this article (10.1186/s13018-017-0685-x) contains supplementary material, which is available to authorized users.

## Background

Chronic lumbar back pain and sciatica are common symptoms of degenerative conditions of the spine that lead to enormous costs to the health care systems of industrialised countries [1–4]. The diagnosis and resulting conservative or operative treatment is based on the patient’s medical history and concerns, physical examination and radiographic imaging, especially X-rays and magnetic resonance imaging (MRI) scans. On the basis of improvements in diagnostic imaging and surgical techniques, therapeutic strategies have become increasingly focused on surgical treatment [5, 6]. Despite all improvements, however, especially in patients with chronic multisegmental lumbar disease and spinal stenosis observed on MRI, clinicians still cannot reliably predict the success of spinal decompression and/or fusion surgery. Since it has been established that neither clinical findings [7, 8] nor radiologic facet joint pathology [9–11] can be used to reliably diagnose a painful facet joint, local targeted infiltrations can be additionally used to temporarily simulate the effect of surgery through local administration of local analgesic and anti-inflammatory agents to the facet joints [12–16].

So far, no consensus has been established about whether radiologic imaging can predict the response to diagnostic or therapeutic facet joint blocks (reviewed by Cohen and Raja (2007)) [17]. In their 2010 study, Stojanonic et al. described a possible association between MRI spinal stenosis and successful extra-articular medial branch block infiltrations. Even though these study results failed to reach statistical significance, this finding might offer an additional perspective on how to consider the effects observed by facet joint infiltrations [18].

In the present study, we aimed to systematically evaluate the quality of different measurement and classification systems for spinal stenosis and facet joint degeneration on MRI for their ability to predict reported pain relief after facet joint infiltration in patients with chronic lumbar back pain. We hypothesised that, as pathological grading increased in MRI scans, pain alleviation would also increase after bilateral facet joint infiltration.

## Methods

### Study design

All patients who had received inpatient gradual diagnostics [12, 13] from 2005 to 2016 for chronic lumbar back pain were screened for inclusion in the study. Inclusion criteria included undergoing a monosegmental facet joint infiltration on the first day of inpatient gradual diagnostics and clearly stated pain relief in percentage (%) for that specific infiltration in the medical documentation. Moreover, the pain level prior to infiltration needed to be clearly documented and a high-quality MRI available before infiltration. Patients were excluded if they had a positive history of lumbar surgery or the presence of artificial implants in the area of interest.

Various measurement and grading techniques were used to evaluate facet joint degeneration, neuroforaminal stenosis or spinal canal stenosis on the MR images. All measurements were performed blinded by the same observer, who was familiar with and had practiced all tested measurement techniques.

Full departmental, institutional and local ethical committee approvals were obtained before commencement of the study (project number 503/2016BO2).

### Infiltration technique and reported pain relief

An analgesic (0.5 ml bupivacaine 0.25%) and a corticosteroid (0.5 ml triamcinolone 10 mg/ml) were injected intraarticularly into the facet joint under fluoroscopic guidance. Patients were then asked on the following day to report the pain relief obtained by the infiltration in terms of percentage (%) of the total pain present before infiltration.

### Measurement technique

Degeneration of the facet joints was classified by using the method of Weishaupt et al. [19, 20], which uses T2 images to evaluate the presence of osteophytes, subchondral cysts, bone erosions and possible joint space narrowing to allocate degeneration grades ranging from 0 to 3. Because infiltrations were performed bilaterally, the higher degeneration grade was used for further statistical analyses.

To stratify stenosis of the spinal canal and the neuroforamen, we performed both qualitative and quantitative techniques. Again, the higher pathological value from both sides was used for further analyses.

**Fig. 1:**
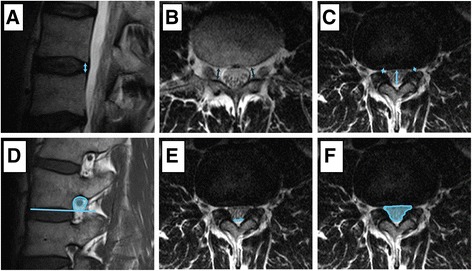
Quantitative measurements: **a** Sagittal T2, **b**, **c**, **e**, **f** axial T2 and **d** sagittal T1 images. **a** Posterior disc height [21]. **b** Neuroforaminal antero-posterior distance at the level where the root can be seen to traverse it [22] and **c** at the level of the intervertebral disc. The blue median arrow shows the sagittal diameter of the dural sac at that level. **d** Cross-sectional area of the neuroforamen [23], **e** ligamentous interfacet distance [26] as a line connecting the ventral joint space of the facet joints between the inner surface of the flaval ligaments and **f** cross-sectional area of the dural sac [27–29] at the level of the intervertebral disc. The lateral margins of the dura in the neuroforaminal area were extrapolated from the images above and below

Quantitative neuroforaminal stenosis measurements:

I. In sagittal T2 images, posterior disc height [21] was measured after the central position of the spinal canal in the axial plane was identified (Fig. 1a).
II. The minimum antero-posterior diameter of the neuroforamen was measured in axial T2 images in two ways: First, it was measured in the axial plane where the root can be seen to traverse it [22] (Fig. 1b). The second measurement was performed at the level where the location of the intervertebral disc was confirmed in sagittal T2 images. If several images were available that met these criteria, the more cranial one was analysed (Fig. 1c).
III. The minimum cross-sectional area of the foraminal zone was measured on sagittal T1 images. As suggested by Sipola et al. [23], the key area of interest was the zone below the pedicle because of the cranial transition of the nerve root in the foramen. Therefore, no space below a line parallel to the lower end plate was included (Fig. 1d).

Using sagittal T1 images, we qualitatively graded neuroforaminal stenosis, as proposed by Lee et al. [24, 25], whereby stages 0–3 are allocated according to the degree of nerve root compression at the narrowest point at the medial margin of the pedicle in the subpedicular zone.

Quantitative spinal canal stenosis measurements:

I. Axial T2 images at the level of the intervertebral disc were used to measure the ligamentous interfacet distance [26]. This distance covers a line connecting the ventral joint space of the facet joints between the inner surface of the flaval ligaments. If two adjacent images were available at the disc level, the narrower distance was measured (Fig. 1e).
II. Antero-posterior constriction was measured as the mid-sagittal diameter of the dural sac at its narrowest level in axial T2 images (Fig. 1f).
III. The smallest cross-sectional area of the dural sac at the infiltrated level was measured in T2 axial images [27–29]. The lateral margins in the neuroforaminal area were extrapolated from the images above and below (Fig. 1f).

We qualitatively graded spinal canal stenosis, as suggested by Schizas et al.,[30] in axial T2 images, whereby categories A1-4 were subsumed as A.

### Imaging

All three Tesla or 1.5 Tesla MRIs were available in digital form and analysed on an Eizo RadiForce RS110 48-cm Class Colour LCD screen (Eizo Nanao Corporation, Hakusan, Ishikawa, Japan) with a centricity PACS Radiology RA1000 workstation (GE Healthcare, Barrington, IL, USA).

### Statistical analysis

Distributions of variables for all parameters were assessed as histograms. Categorical variables are described as absolute frequencies. Depending on normality, data are reported as mean (standard deviation) or median (minimum-maximum). Differences between two groups were calculated by *t* test for independent samples and Mann-Whitney *U* test. To evaluate the association of reported pain relief and imaging findings, we calculated Kendall’s tau correlation coefficient. For further analyses, pain relief was additionally categorised into three groups: no (below 30%), good (30–74%) and excellent (75–100%) pain relief.

All reported *p* values have a two-tailed significance level of alpha = 0.05. No adjustment for multiple testing was performed. Graphic illustration of the results was performed by using bar diagrams, boxplots and scatterplots. Statistical analysis was conducted with IBM SPSS version 22.

## Results

**Fig. 2:**
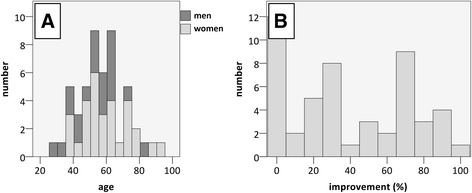
**a** Histogram displaying patient age with a peak around 55 years. **b** Heterogeneous improvement after facet joint infiltration ranging from 0 to 100%, with a median improvement of 30%

In total, 50 patients met all inclusion criteria (30 women and 20 men). The median age was 57 (28–95) years (Fig. 2a). From the time of MRI to infiltration, the median elapsed time was 54 (0–295) days. The preinfiltration pain level on a numeric rating scale was 4 (3–9), with a median improvement after facet joint infiltration of 30% (0–100%) (Fig. 2b). When we allocated patients according to their reported pain relief, 27 showed no improvement (0–30%), 16 reported good improvement (31–75%) and 7 reported excellent improvement (> 75%). Twenty-four patients received infiltrations on facet joints L4/5, 22 on L5/S1 and 2 on L2/3 and L3/4.

**Fig. 3:**
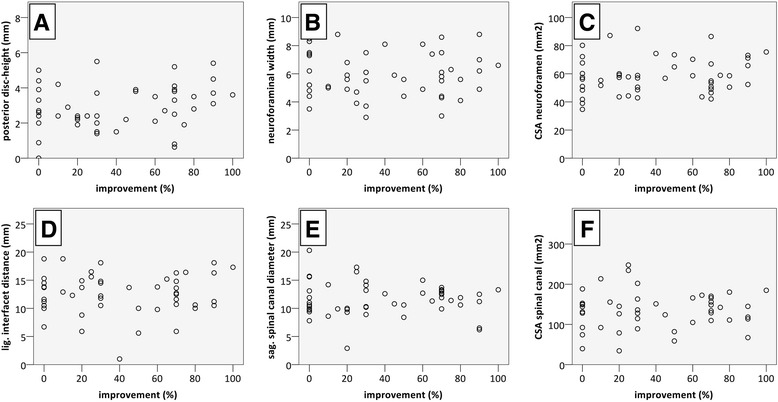
Improvement obtained by infiltration and radiomorphometric measurements described as scatterplots: posterior disc height [21] (**a**), neuroforaminal width [22] (**b**), cross-sectional area of the neuroforamen [23] (**c**), ligamentous interfacet distance [26] (**d**), cross-sectional area of the spinal canal (**e**) and sagittal spinal canal diameter [26, 55–57] (**f**). No relevant correlation could be observed. CSA cross-sectional area, lig. ligamentous, sag. sagittal

**Fig. 4:**
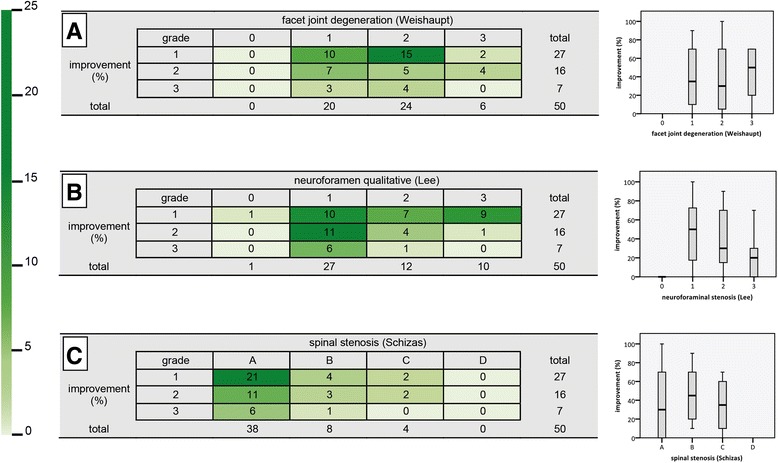
Correlation of observed improvement by infiltrations with qualitative facet joint degeneration and spinal stenosis classifications in the form of heat maps (left column) and boxplots (right column). **a** Facet joint degeneration (Weishaupt [20]), **b** neuroforaminal stenosis (Lee [25]) and **c** spinal stenosis (Schizas [30]). Pain relief obtained by the infiltrations is grouped into three categories: 1, no improvement (below 30%); 2, good (30–74%); and 3, excellent (75–100%) improvement. Colour intensity in the heat maps is shown according to absolute frequencies

No relevant connection could be observed between reported improvement after infiltration and MRI findings for any of the analysed parameters (Table 1, Figs. 3 and 4).

**Table 1:** Correlation analysis of radiomorphometric measurements or qualitative classifications and pain relief after facet joint injection

Variable	Kendall’s tau	*p* value
Qualitative measurements		
Facet joint degeneration (Weishaupt) [20]	− 0.020	0.866
Neuroforaminal stenosis (Lee) [58]	− 0.190	0.103
Spinal canal stenosis (Schizas) [30]	0.036	0.767
Quantitative measurements		
Posterior disc height [21]	0.133	0.195
Neuroforaminal antero-posterior distance [22]	− 0.007	0.946
Neuroforaminal cross-sectional area [23]	0.085	0.402
Ligamentous interfacet distance [26]	− 0.026	0.799
Minimum sagittal antero-posterior diameter of the spinal canal	0.030	0.767
Minimum cross-sectional area of the spinal canal [27–29]	− 0.022	0.826

Even when we performed a subgroup analysis between those patients with maximum improvement (100%, *n* = 7) and those with no improvement at all (0%, *n* = 12), no significant difference was notable (Table 2). The original study data are available as Additional file 1.

**Table 2:** Analysis of patients with maximum pain relief and no response

Variable	0% pain relief (*n* = 18)	100% pain relief (*n* = 11)	*p* value
Infiltrated motion segments			0.711^a^
Qualitative measurements			
Facet joint degeneration (Weishaupt) [20]			0.837^a^
Neuroforaminal stenosis (Lee) [58]			0.432^a^
Spinal canal stenosis (Schizas) [30]			0.902^a^
Quantitative measurements			
Posterior disc height [21]	2.8 (2.1)	3.8 (0.9)	0.266^b^
Neuroforaminal antero-posterior distance [22]	6.3 (1.6)	6.2 (1.5)	0.860^b^
Neuroforaminal cross-sectional area [23]	58.3 (16.9)	63.9 (10.1)	0.439^b^
Ligamentous interfacet distance [26]	13.5 (4.1)	13.4 (3.6)	0.973^b^
Minimum sagittal antero-posterior diameter of the spinal canal	12.1 (3.5)	10.3 (2.8)	0.260^b^
Minimum cross-sectional area of the spinal canal [27–29]	155.3 (81.3)	131.4 (41.7)	0.482^b^

No difference in motion segments between those two groups was observed. Values are reported as means (standard deviation)

^a^Mann-Whitney *U* test

^b^ *t* test for independent samples

## Discussion

When we analysed the relationship between reported pain relief after fluoroscopy-guided facet joint infiltrations and qualitative or quantitative radiomorphometric parameters, no relevant connection could be found. In 2007, Gorbach et al. described a lack of correlation between success of facet joint infiltration and imaging grading of facet joint degeneration when using the system suggested by Weishaupt [31]. In 2010, Stojanovic et al. also described only a weak correlation of MRI facet joint hypertrophy and a positive response to diagnostic medial branch blocks [18].

With further improvements in imaging techniques that allow detailed visualisation of spinal structures, radiographic findings are increasingly considered to be solid evidence, similar to laboratory test results or histopathological findings. This anticipated confidence might dispose surgeons to largely base their recommendations for treatment strategies on such imaging. The correlation between radiological and clinical findings to distinguish between symptomatic and asymptomatic patients is, however, limited and unreliable for all common modalities such as X-ray, computed tomography, MRI scan or single-photon emission computed tomography (SPECT) scan [32–35]. This applies to both facet joint degeneration [36, 37] and spinal stenosis, independent of whether quantitative [38, 39] or qualitative stenosis classifications [40] are used. Clear correlations are usually described only for different parameters of the same technique: the occurrence of intraarticular fluid of the facet joints, for example, is known to increase with degenerative spondylolisthesis [41, 42] and appears to be associated with lumbar instability [42, 43]. Of note, this observation does not describe a clinical symptom. In addition, when examining the literature on that topic critically, one must not ignore that reported positive correlations between two phenomena such as clinical and radiologic findings bear an intrinsic publication bias, so that it is only after the initial euphoria in the scientific community that clear clinical relevance can be established.

Efforts nevertheless continue in order to improve diagnostic predictability of imaging techniques: although the supine MRI technique still predominates, upright imaging might improve the results because of the weight-bearing condition. Still, it does not provide dynamic, but only static information. Another difficulty with the correct interpretation of imaging results occurs in circumstances in which adaptational processes are difficult or even impossible to visualise. This can especially be noted when the clinical presentation of acute minor nuclear prolapses is compared with those of elderly patients with a long history of what is, in many cases, asymptomatic severe spinal stenosis. One explanation for such differences might be the triggered inflammatory processes that lead to swelling or intraarticular synovial fluid collection, which in peripheral joints is easily recognisable clinically, whereas the zygapophyseal joints do not offer such a clinical feature. In addition, fluid production in the facet joints seen on MRI is presently interpreted as a sign of instability [41–43] rather than a sign of inflammation. Perhaps the activation of the degenerative stage could be visualised by imaging of metabolic processes, using, for example, SPECT-CT or positron emission tomography-CT [44]. It is still a matter of discussion whether the response to infiltration and the prognostic value of facet joint degeneration in these imaging modalities is increased compared with those for MRI. Furthermore, the power of imaging often lies in its ability to rule out other differential diagnoses, such as fracture, infection or neoplasm, rather than to prove a symptomatic condition.

In compensation for this diagnostic imaging gap, image-guided diagnostic facet joint infiltrations have been established, for which evidence is considered strong (level II) to isolate the facet joint as a pain generator [45]. To the best of our knowledge, a study evaluating the prognostic value of diagnostic facet joint infiltrations for the outcome of spinal fusion surgery is still lacking and thus, preoperative diagnostic infiltrations remain an eminence-based procedure. First data are, however, available on the prognostic value of facet joint infiltrations before lumbar facet radiofrequency denervation that show that a correct prognosis was made in about 60–70% of analysed patients [46]. The role of such infiltrations has nonetheless been recently questioned. Schütz et al. [47] performed a triple cross-over study to investigate the effects obtained by diagnostic facet joint infiltrations compared with those by placebo and sham infiltrations. They found no relevant difference between the three modalities and thus questioned the diagnostic value of medial branch blocks. Indeed, a high false positive rate for facet joint infiltrations is described in the literature [48]. When the data are examined closely, however, one general problem with most studies on the topic must be recognised: patients with chronic lumbar back pain usually do not present with monosegmental problems but rather with multisegmental changes, which in a chronic form must be considered a complex syndrome compared with monosegmental facet joint pathology. This consideration also includes the locus of nociception: whereas nociception in facet joint syndrome has been suggested to originate in the synovial membrane, hyaline cartilage, bone or fibrous capsule of the facet joint [17], in chronic conditions, structures other than the facet joints themselves, such as myofascial trigger points or even reactive overexcitability of nociceptive neurons in the central nervous system [49], can provide the nociceptors. Moreover, dual innervation of the facet joint with overlapping zones of referred pain [50–52] makes medial branch blocks inadequate for diagnosis of a single motion segment’s facet joints. In contrast to intraarticular injections (as in the present study), medial branch blocks also seem to anaesthetise not just the joint but also the muscles, ligaments and periosteum that they innervate [53]. Although this does not argue against performing medial branch blocks per se, this information needs to be considered when interpreting data from the literature.

Given the uncertainty surrounding both the interpretability of MRI findings and the diagnostic value of facet joint infiltrations, it is clear that future studies should first concentrate on patients with monosegmental problems. Only when a clear determination of sensitivity, specificity, validity and reliability has been obtained can multisegmental problems be addressed. In view of the given uncertainty of both modalities, the results obtained in the present study could be interpreted in two ways. First, it is possible that either of these techniques or both are completely useless. Second, the results could imply that the information that both modalities provide is complementary. There is no completely reliable gold standard with which to compare a diagnostic test (or injection) when the absence of pain is the end point [54]. It is therefore clear that, for the time being, not only the sum of findings from one modality such as imaging, but also a thorough clinical examination, medical history and—in select cases—infiltration results will allow the development of a solid therapeutic recommendation.

### Study limitations

All infiltrations were guided by fluoroscopy. Nevertheless, it is possible that some of the infiltrations were not applied intraarticularly, but periarticularly. The clear difference between the effects obtained by intraarticular and periarticular infiltrations is yet to be demonstrated. A general critical feature of most studies addressing patients with chronic lumbar back pain is the mostly multisegmental underlying pathology that makes it difficult to analyse monosegmental effects. A crucial consideration for the correct interpretation of our findings is that the results described apply only to chronic conditions.

## Conclusion

Although imaging results are frequently considered as hallmarks of disease by specialists to plan their future treatment strategy, a clear correlation of symptoms and imaging results is not yet possible with current techniques. The prognostic value of facet joint infiltrations for surgical outcome has also recently been questioned. Our results show an absolute lack of correlation between imaging results with MRI and effects obtained by targeted facet joint infiltration. In view of the trend in evidence-based medicine to provide medical algorithms, our findings underline the continuing need for individualised spine medicine that, along with imaging techniques or targeted infiltrations, includes diagnostic dimensions such as good patient history and clinical examination.

## Additional file

Additional file 1: Original study data. (XLSX 23 kb)

## Abbreviations

MRI: Magnetic resonance imaging
SPECT: Single-photon emission computed tomography
